# Evaluation of regional cerebral glucose metabolism in patients with malignant lymphoma of the body using statistical image analysis

**DOI:** 10.1007/s12149-014-0890-1

**Published:** 2014-08-12

**Authors:** Masanari Nonokuma, Yasuo Kuwabara, Koichi Takano, Kazuo Tamura, Kenji Ishitsuka, Kengo Yoshimitsu

**Affiliations:** 1 Department of Radiology, Fukuoka University Hospital, Fukuoka, Japan; 2 Department of Oncology, Hematology and Infectious Diseases, Fukuoka University Hospital, 7-45-1 Nanakuma, Jonan-ku, Fukuoka, 814-0180 Japan

**Keywords:** Malignant lymphoma, FDG-PET/CT, Paraneoplastic syndrome, Regional cerebral glucose metabolism, Statistical image analysis

## Abstract

**Objectives:**

The aim of this study was to clarify the characteristics of regional cerebral glucose metabolic abnormalities in patients with malignant lymphoma of the body using statistical image analyses. Post-therapeutic changes in cerebral glucose metabolism were also evaluated.

**Methods:**

The subjects consisted of 30 patients, including 16 patients with diffuse large B-cell lymphoma and 14 patients with other types of lymphoma. Patients with primary cerebral lymphoma were excluded from this study. All patients underwent CT and whole-body FDG-PET scans, including 4-min brain scans using a dedicated PET/CT scanner during both the pre- and post-treatment periods. The whole-body scans started 60 min after the administration of 185 MBq of FDG, after which the brain data were extracted from whole-body data. The degree of regional cerebral glucose metabolism was evaluated on a voxel-by-voxel basis using statistical parametric mapping (SPM). The total tumor glycolytic volume of the body was measured using a separate workstation. The normal control subjects were 12 persons who underwent medical check with FDG-PET/CT and had no lesions suggesting malignant tumor.

**Results:**

The level of regional cerebral glucose metabolism decreased in association with an increase in the total glycolytic volume in the bilateral frontal and parietal cortices. After chemotherapy, the statistical image analysis demonstrated an interval recovery of the cerebral glucose metabolism of the bilateral parietal and occipital cortices in the good responders, whereas there were no significant differences observed in regional cerebral glucose metabolism between the pre- and post-treatment images in the poor responders. Comparison between normal control subjects and patients with pre-treatment lymphoma also showed that the regional cerebral glucose metabolism decreased in the parieto-occipital cortices in patients with lymphoma compared to normal control subjects.

**Conclusions:**

We demonstrated that patients with malignant lymphoma of the body exhibited abnormal regional cerebral glucose metabolism, which improves after chemotherapy. Although the mechanism underlying the reduction of cerebral glucose metabolism remains unclear, our findings indicate the functional alternation and/or subclinical damage of the brain in patients with malignant lymphoma.

## Introduction

It is well known that some of the cancer patients exhibit neuropsychiatric symptoms without apparent involvement of the tumor with the central nervous system, so-called paraneoplastic syndrome (PNS) [1]. We experienced a patient with malignant lymphoma of the body who displayed bilateral fronto-parieto-occipital hypometabolism without apparent abnormalities on brain MRI. Regarding cerebral glucose metabolism in patients with malignant lymphoma, it has previously been reported that the global FDG uptake of the brain decreases in correlation with the total glycolytic volume (TGV) of the body [2]. However, there are no descriptions regarding regional changes in cerebral glucose metabolism. We retrospectively evaluated regional glucose metabolism in patients with malignant lymphoma of the body and attempted to elucidate the characteristics of regional cerebral glucose metabolic abnormalities using statistical image analysis. Post-therapeutic changes in the cerebral glucose metabolism were also evaluated.

## Materials and methods

The subjects consisted of 30 patients (30–86 years old, average: 56.4 years old, 15 females and 15 men) who underwent dedicated FDG-PET/CT for the pre-treatment staging of malignant lymphoma of the body, including 16 patients with diffuse large B-cell lymphoma, five patients with natural killer cell T-cell lymphoma, three patients with follicular lymphoma, three patients with Hodgkin’s lymphoma, two patients with T-cell lymphoma and one patient with intravascular large B-cell lymphoma. The patients were classified as having stage I (four patients), stage II (eight patients), stage III (eight patients) and stage IV (10 patients) disease according to the revised Ann Arbor classification proposed by Cotswold [3]. The therapeutic response was a complete response (CR) in 19 patients, partial response (PR) in eight patients and progressive disease (PD) in three patients based on the International Working Group response criteria [4]. All patients underwent FDG-PET/CT during both the pre- and post-treatment periods. The treatment regimens included chemotherapy (rituximab, cyclophosphamide, doxorubicin, vincristine and prednisone; R-CHOP: 16 patients, cyclophosphamide, doxorubicin, vincristine, and prednisone; CHOP: three patients, adriamycin, bleomycin, vinblastine, and dacarbazine; ABVD: two patients and dexamethasone, etoposide, ifosfamide and carboplatin; DeVIC: two patients), radiation therapy (one patient) and surgical therapy (one patient). Five of the 30 patients underwent brain MRI. The normal control subjects were 12 persons (42–80 old years, average: 62.0 years old, 8 females and 4 men) who underwent medical checkup with FDG-PET/CT and had no lesions suggesting malignant tumor. Patients with primary cerebral lymphoma or apparent cerebral abnormalities, such as cerebral infarcts more than 10 mm in diameter on CT or MRI, were excluded from this study. Subjects with apparent miss-registration between the FDG-PET and CT images due to head movement during the examinations were also excluded based on a visual inspection of the integrated PET/CT images. Subjects with a blood sugar level over 120 mg/dl were excluded to prevent the effects of hyperglycemia on the FDG brain uptake. The blood sugar levels of 30 patients ranged from 68 to 119 mg/dl (mean ± SD: 92.1 ± 13.1 mg/dl) in the pre-treatment period and from 65 to 119 mg/dl (mean ± SD: 92.1 ± 12.5 mg/dl) in the post-treatment period. Those of controls subjects were from 63 to 116 mg/dl (mean ± SD: 92.3 ± 14.9 mg/dl). No subjects received psychotropic drugs, such as sedatives, at the time of the PET studies. The interval between the pre- and post-treatment FDG-PET/CT scans ranged from 2.9 to 20 months (mean ± SD: 7.9 ± 3.6 months).

### FDG-PET/CT

After fasting for at least 4 h, each subject received an intravenous infusion of 185 MBq of FDG over 2 min while sitting in a chair, then was moved to a semi-dark room and placed in the supine position on a reclining seat for 60 min. The PET/CT scanner used in this study was the Aquiduo (Toshiba/Siemens Co. Ltd). First, non-contrast whole-body CT data were obtained in helical mode with an X-ray tube voltage peak of 120 keV, a flexible X-ray tube current of 50–400 mA, a field of view of 500 × 500 mm, and a reconstructed slice thickness of 2 mm. The whole-body PET scan was started 60 min after the administration of FDG. The subjects were asked to breathe normally during PET acquisition, and FDG-PET data were collected in the three-dimensional imaging mode for 2 min at each position from foot to head; thus, the brain data were obtained from 76 to 80 min after the administration of FDG. The PET data were reconstructed using a CT transmission map for attenuation correction with the ordered-subsets expectation maximization algorithm (4 iterations, 14 subsets) and a Gaussian Filter (FWHM = 7.0 mm) and displayed in a 128 matrix (pixel size = 3.9 × 3.9 mm with a slice thickness of 2.0 mm).

### Data analysis

The brain data were extracted from the whole-body data, and brain images in the Digital Imaging and Communications in Medicine (DICOM) format were converted to the analyze format using MRlcro (Chris Rorden’s MRIcro, Copyright 1999–2005, all rights reserved.) on a workstation, after which clipping and centering of the images were performed. Anatomical normalization into the MNI standard space (Montreal Neurological Institute, McGill University, Montreal, Canada) was performed with the Statistical Parametric Mapping 2 software package (SPM2, Welcome Department of Cognitive Neurology, London, UK) and FDG template running under MATLAB R2009a (Math Works Inc., Sherborn, MA, USA). Spatially normalized images were smoothed via convolution using an isotopic Gaussian kernel with a 12-mm full-width half-maximum. Statistical comparisons were made on a voxel-by-voxel basis using SPM8 to generate SPM (t) maps. A multiple regression analysis was used to evaluate the correlations between the degree of regional cerebral glucose metabolism and other parameters, including TGV and the clinical stage. The two-tailed paired *t* test was used to compare the brain images between the pre- and post-treatment examinations. The resulting maps of *t* statistics were displayed at height thresholds of *p* < 0.005 (paired *t* test), uncorrected for multiple comparisons between the pre-treatment and post-treatment patients, and displayed at height thresholds of *p* < 0.02 (non-paired *t* test), uncorrected for multiple comparisons between normal control subjects and patients with lymphoma. The extent threshold of 200 voxels was also used to determine the statistical significance of the cluster.

### Measurement of the total glycolytic volume

The TGV of the body was measured using a separate workstation (Virtual Place, AZE, Japan). The tumor volumes were measured semiautomatically using ROIs set on the whole-body images. A threshold was determined at the level of 2.5 of SUV [5], and areas with a higher FDG uptake were automatically extracted, while the areas of physiological FDG uptake were manually eliminated. Finally, both the total volume of the areas over the threshold and the mean SUV were measured. The TGV was calculated according to the following equation: TGV = tumor volume × mean SUV [2]. The study protocol was approved by the Independent Ethics Committee/Institution Review Board of Fukuoka University Hospital.

## Results

**Fig. 1:**
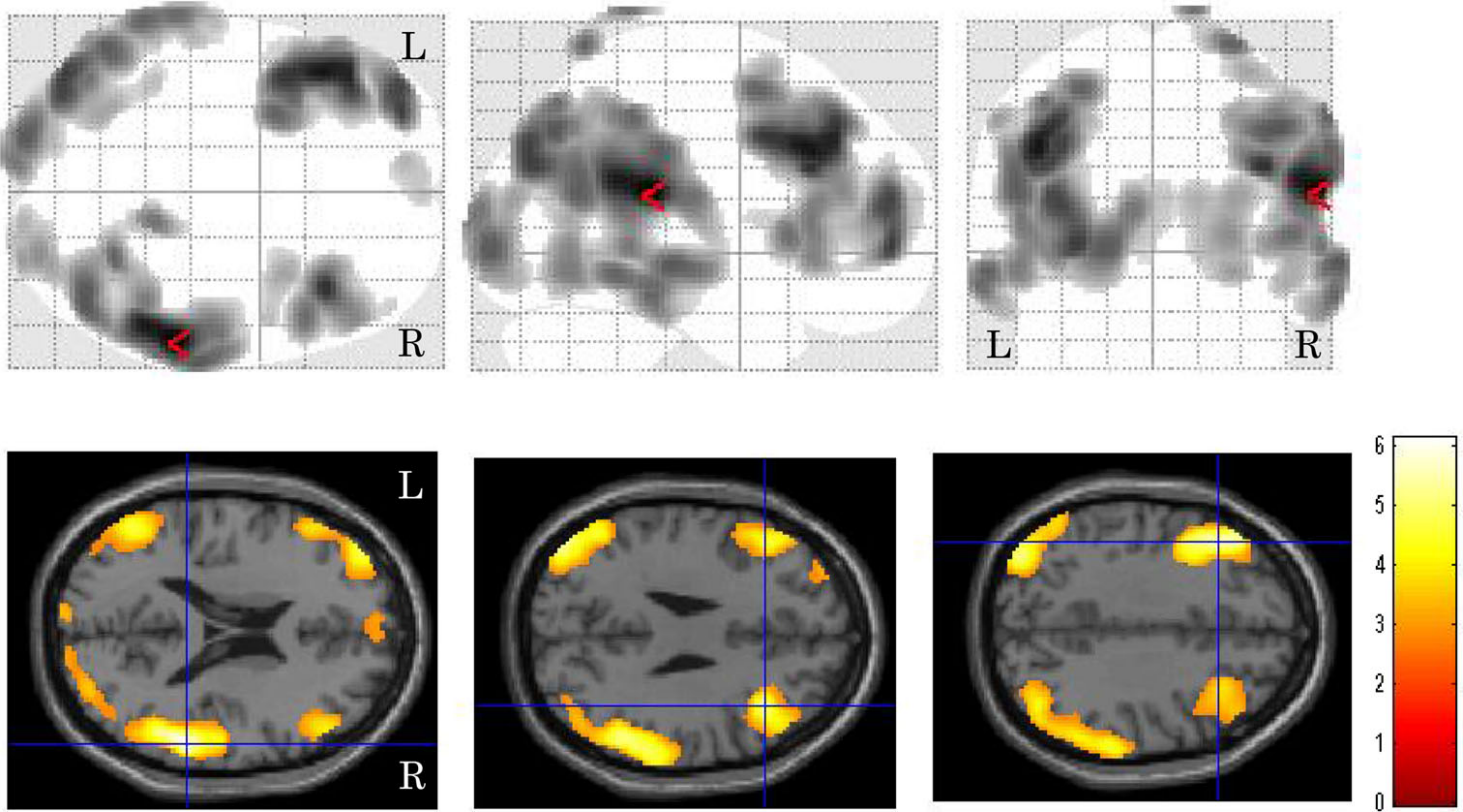
Images of brain regions showing a significant correlation between regional cerebral glucose metabolism and the TGV. The presence of bilateral frontal, temporal and parietal glucose metabolism was negatively correlated with the TGV

**Fig. 2:**
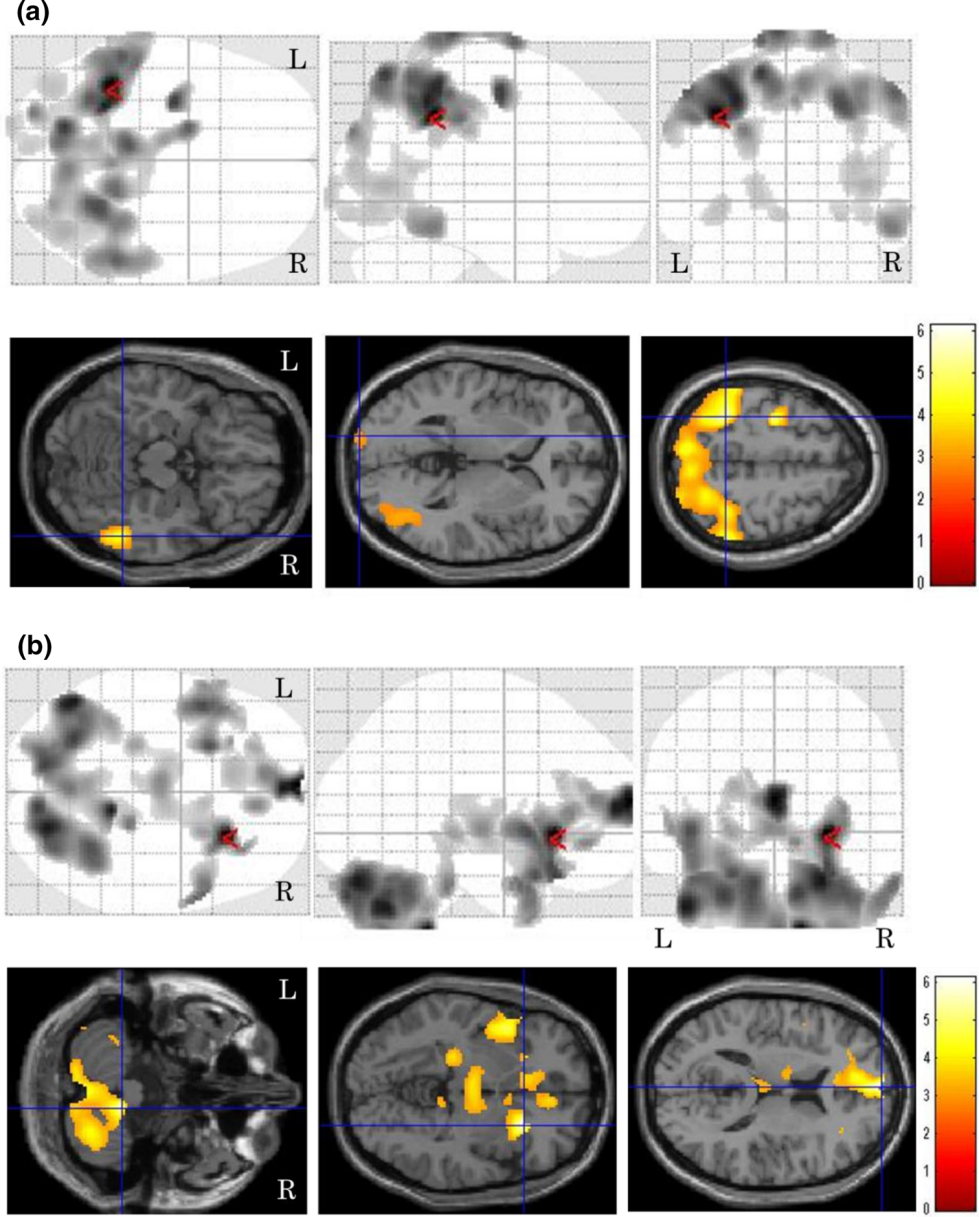
Images of brain regions showing significant differences in cerebral glucose metabolism between the pre- and post-treatment examinations. The level of cerebral glucose metabolism was significantly increased in the bilateral parietal and occipital regions after treatment (**a**) and decreased in the bilateral cerebellar hemisphere, right putamen, bilateral insula and bilateral anterior cingulate regions (**b**)

**Fig. 3:**
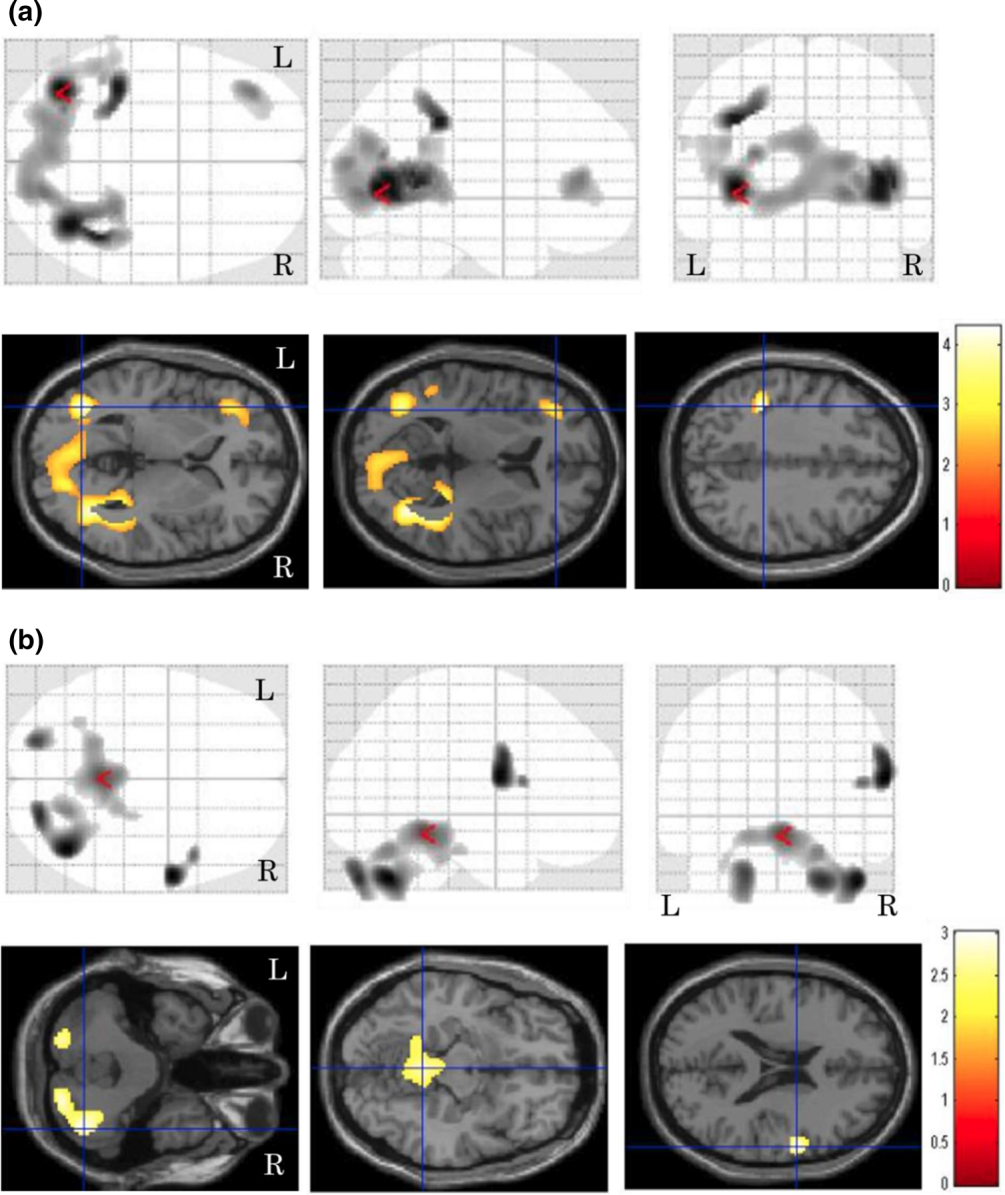
Images of brain regions showing significant differences in cerebral glucose metabolism between pre-treatment patients and normal control subjects. The level of cerebral glucose metabolism in patients with lymphoma was significantly decreased in the bilateral temporal and left parietal and bilateral occipital regions compared to that in the normal control subjects (**a**), while it increased in the right frontal region and bilateral cerebellar hemispheres (**b**)

A significant negative correlation was observed between the average cerebral SUV and the TGV (*y* = −0.0001*x* + 6.25, *R* ^2^ = 0.22, *p* < 0.001). The average cerebral SUV was also correlated with the clinical stage (*y* = −0.57*x* + 7.34, *R* ^2^ = 0.18, *p* < 0.001). The correlation between local cerebral glucose metabolism and the TGV is shown in Fig. 1. The statistical image analysis demonstrated that the bilateral frontal temporal and parietal glucose metabolism was negatively correlated with the TGV. The brain areas showing peak coordinates are summarized in Table 1. Tables 2 and 3 present the areas with significant differences in regional cerebral glucose metabolism between the pre- and post-treatment examinations in the patients with a therapeutic response of CR. After chemotherapy, the degree of regional cerebral glucose metabolism significantly improved in the bilateral parietal and occipital (Fig. 2a) and decreased in the bilateral cerebellar hemisphere, right putamen, bilateral insula and bilateral anterior cingulate regions (Fig. 2b). The regional cerebral glucose metabolism in pre-treatment patients decreased in the left parietal and bilateral occipital regions compared to that in normal control subjects (Fig. 3a), and it increased in the right frontal region and bilateral cerebellar hemispheres (Fig. 3b). The brain areas showing peak coordinates are summarized in Tables 4 and 5. There were no significant changes in regional cerebral glucose metabolism between the pre-and post-chemotherapy examinations in the patients with a therapeutic response of PR or PD.

**Table 1:**
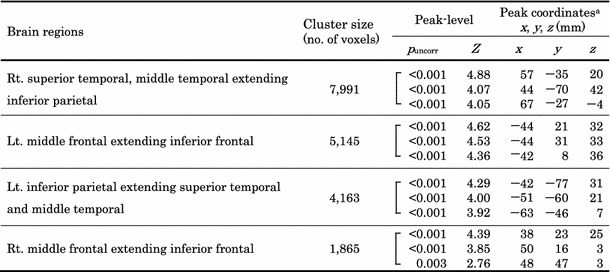
Location of brain areas showing a negative correlation between cerebral glucose metabolism and the total glycolytic volume (TGV)

*p* < 0.005, uncorrected for multiple comparisons

^a^ Coordinated in the Talairach space

**Table 2:**
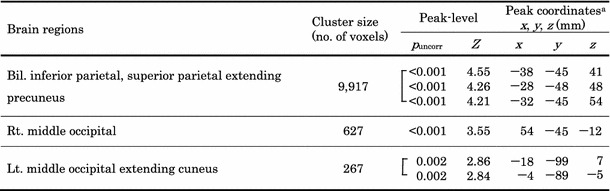
Locations of brain areas showing an interval increase in cerebral glucose metabolism after therapy in patients with a therapeutic response of CR

*p* < 0.005, uncorrected for multiple comparisons

^a^ Coordinated in the Talairach space

**Table 3:**
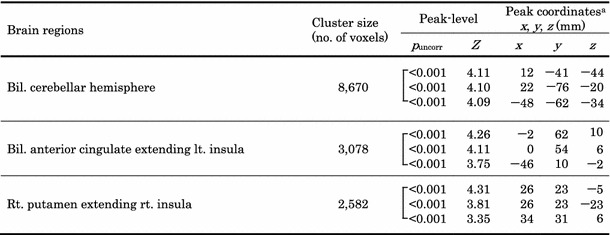
Locations of brain areas showing an interval decrease in cerebral glucose metabolism after therapy in patients with a therapeutic response of CR

*p* < 0.005, uncorrected for multiple comparisons

^a^ Coordinated in the Talairach space

**Table 4:** Locations of brain areas showing an interval increase in cerebral glucose metabolism between the normal groups with patients examinations

Brain regions	Cluster size (no. of voxels)	Peak-level		Peak coordinates^a^ *x*, *y*, *z* (mm)		
		*p*_uncorr_	*Z*	*x*	*y*	*z*
Bil. middle occipital	5,509	<0.02 <0.02	3.88 3.70	−38 −38	−68 −35	5.2 37
Lt. inferior parietal	5,509	<0.02	3.77	38	−62	8.6
Lt. middle frontal	380	<0.02	2.87	−36	39	5.4

*p* < 0.02, uncorrected for multiple comparisons

^a^ Coordinated in the Talairach space

**Table 5:** Locations of brain areas showing an interval decrease in cerebral glucose metabolism between the normal groups with patients examinations

Brain regions	Cluster size (no. of voxels)	Peak-level		Peak coordinates^a^ *x*, *y*, *z* (mm)		
		*p*_uncorr_	*Z*	*x*	*y*	*z*
Bil. vermis	1,390	<0.02	2.55	0	−43	−8.9
Lt. cerebellar hemisphere	1,390	<0.02	2.26	18	−33	−22
Lt. middle frontal	380	<0.02	2.87	−36	39	5.4
Rt. inferior frontal	364	<0.02	2.84	59	0	18
Rt. cerebellar hemisphere	339	<0.02	2.60	−20	−81	−27

*p* < 0.02, uncorrected for multiple comparisons

^a^ Coordinated in the Talairach space

### Case presentation

**Fig. 4:**
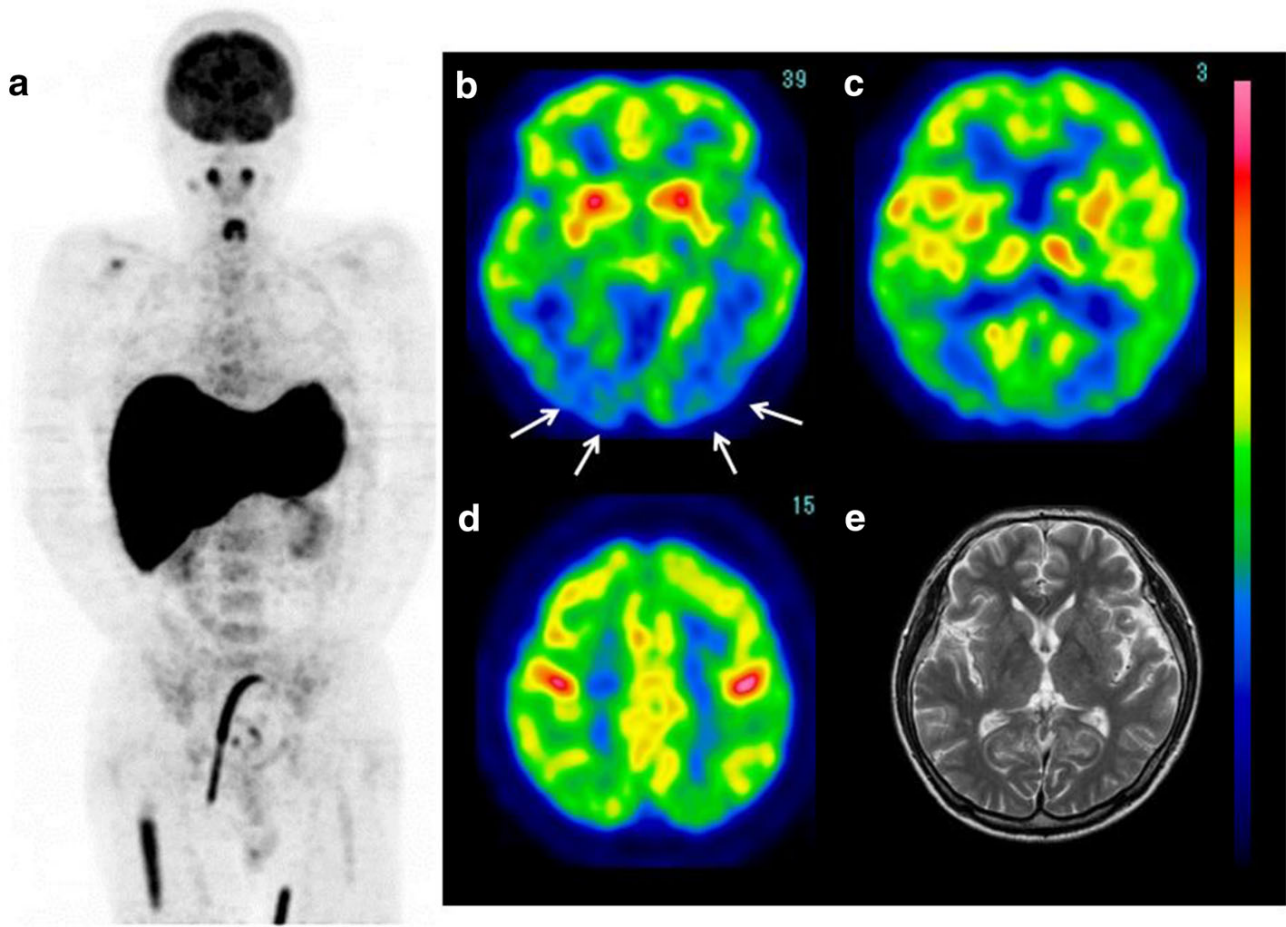
(Case 1: diffuse large B-cell lymphoma, intravascular lymphoma) FDG-PET images of the whole body and brain. Whole-body FDG-PET showed a high uptake in the liver and bilateral femoral bone marrow (**a**). Brain FDG images demonstrated a reduction in glucose metabolism in the bilateral parieto-occipital regions (**b**–**d**). MRI showed no abnormalities in the brain (**e**)

**Fig. 5:**
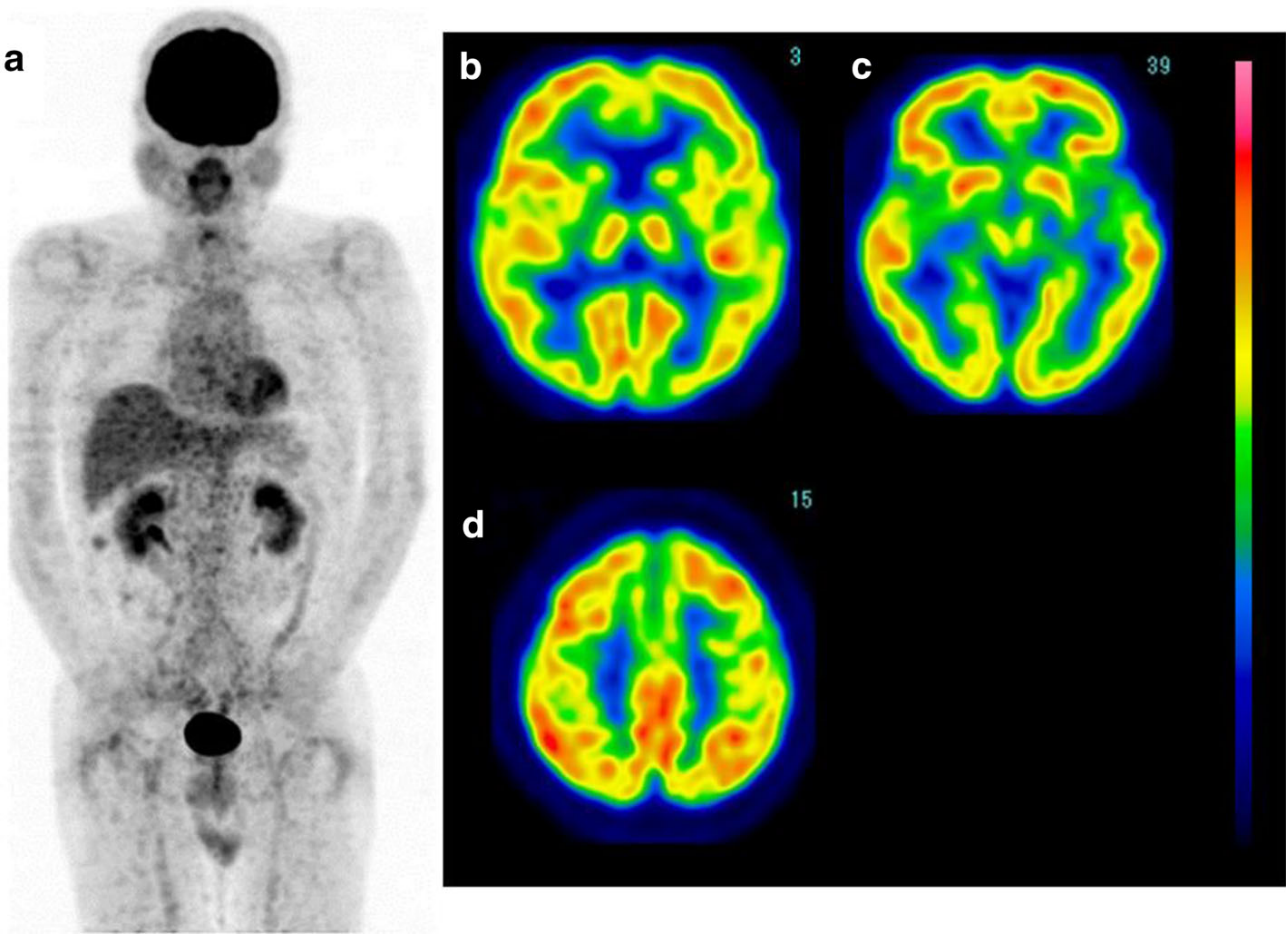
After chemotherapy, the abnormal FDG uptake in the liver and bone marrow disappeared (**a**) and the cerebral glucose metabolism improved (**b**–**d**)

*Case 1* A 59-year-old male was admitted to Fukuoka University Hospital for an examination of the cause of a persistent high fever lasting for 3 weeks in addition to severe liver dysfunction. There were no neurological deficits; however, the patient reported a feeling of dullness in his head. FDG-PET revealed a high FDG uptake in the liver and right femoral bone marrow (Fig. 4a), as well as bilateral parieto-temporal-occipital hypometabolism (Fig. 4b–d). The cerebral glucose metabolism was relatively preserved in the bilateral primary sensory motor cortices and cerebellum. The patient’s blood sugar level was 96 mg/dl. Subsequent brain MRI did not show any abnormalities (Fig. 4e). Therefore, a diagnosis of intravascular lymphoma (IVL) was highly suspected. A needle biopsy confirmed a diagnosis of IVL of the liver (diffuse large cell type). Following the administration of chemotherapy with prednisolone (PSL), etoposide (VP-16) and R-CHOP, the patient’s clinical symptoms and laboratory data markedly improved. The feeling of dullness in his head also disappeared after treatment. Post-treatment FDG-PET/CT revealed no areas of abnormal uptake in the liver or bone marrow (Fig. 5a), and no apparent hypometabolism was observed in the brain (Fig. 5b–d). The patient’s blood sugar level was 91 mg/dl at the time of the second PET study.

### Case presentation

**Fig. 6:**
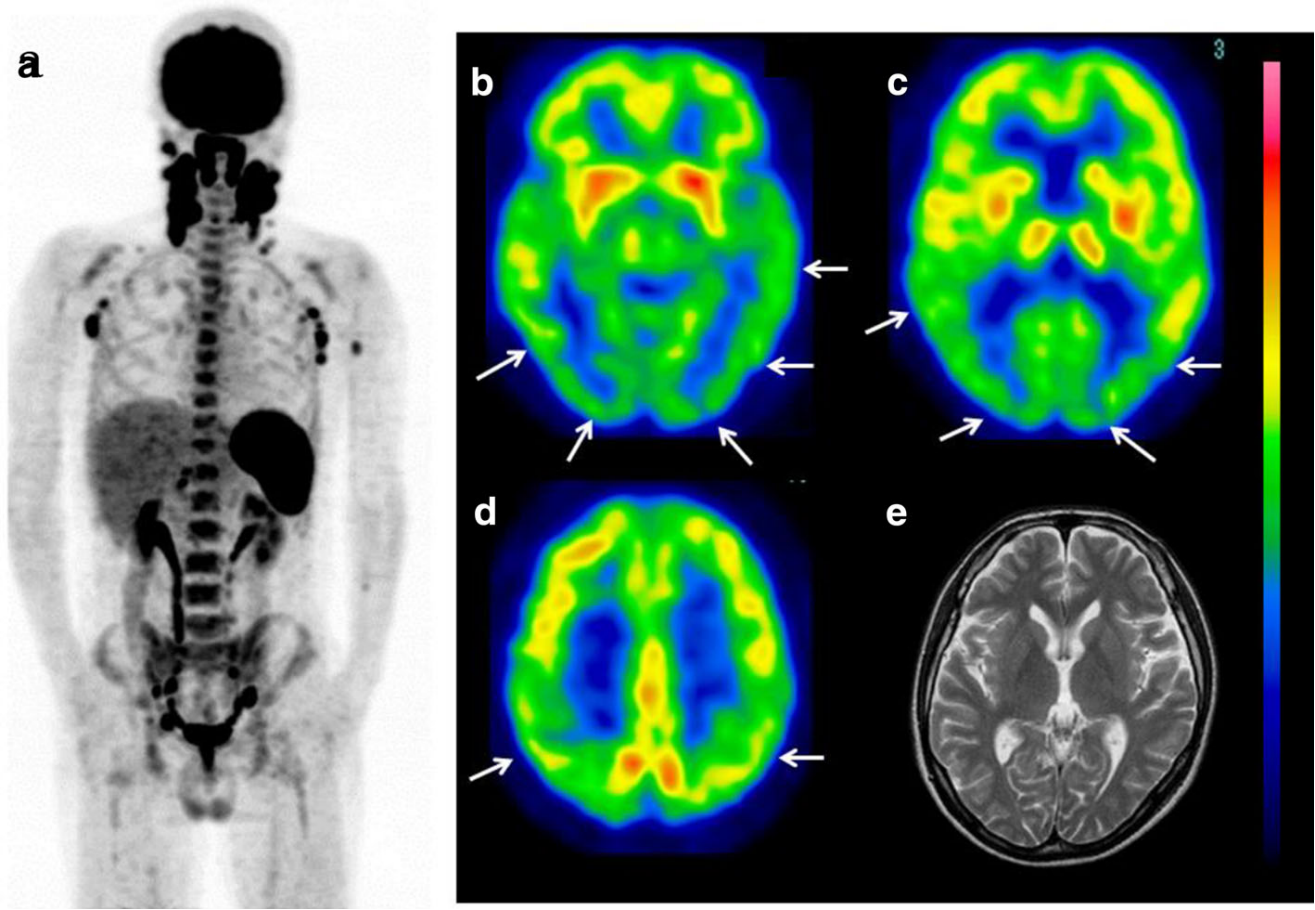
(Case 2: T-cell lymphoma). FDG-PET images of the whole body and brain. Whole-body FDG-PET showed a high uptake in the systemic lymph nodes, spleen and bone marrow (**a**). Brain FDG images demonstrated a reduction in glucose metabolism in the bilateral parieto-occipital regions (**b**–**d**). MRI showed no abnormalities in the brain (**e**)

**Fig. 7:**
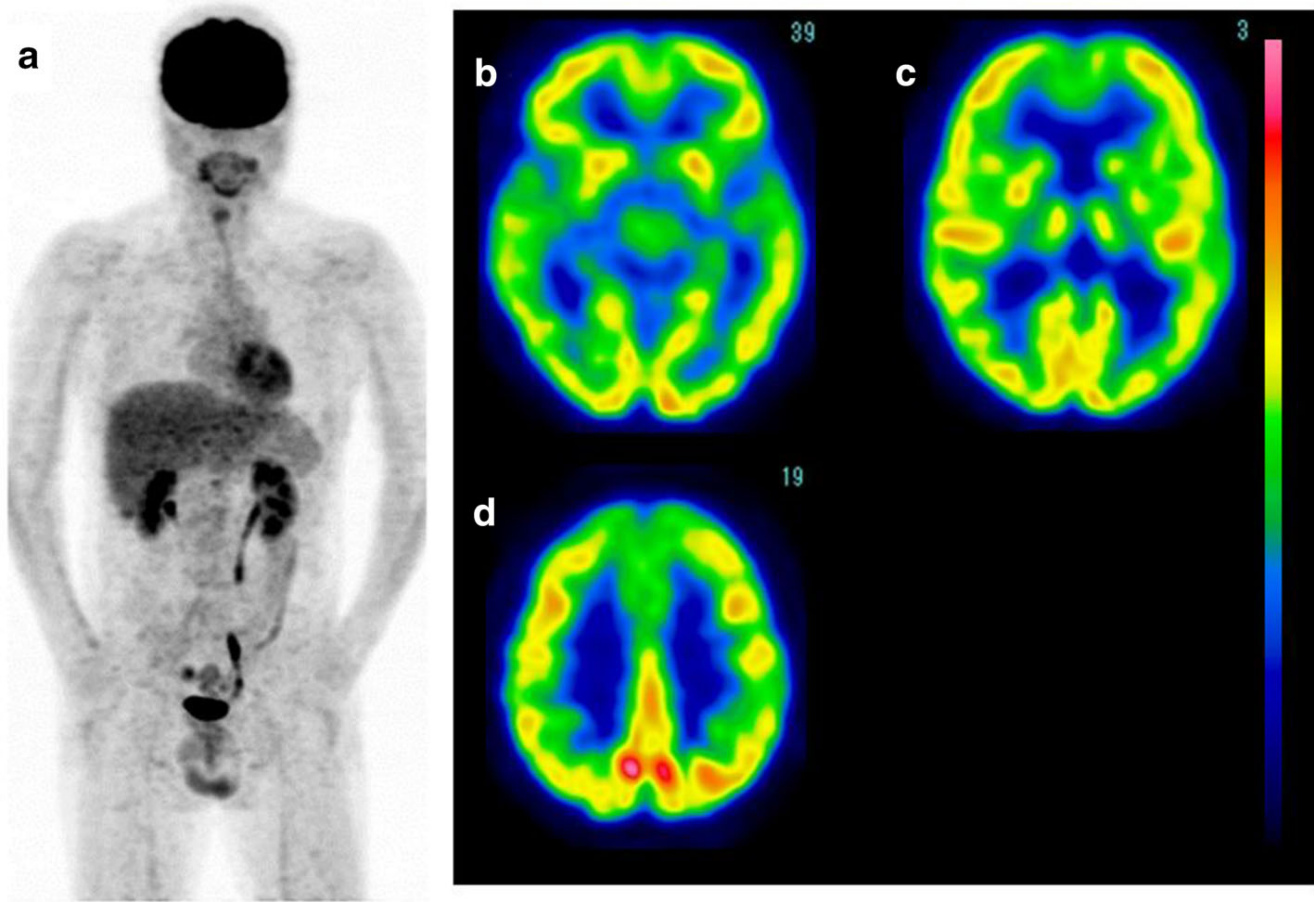
After chemotherapy, the abnormal FDG uptake in these areas completely disappeared (**a**) and the cerebral glucose metabolism improved (**b**–**d**)

*Case 2* A 35-year-old male complained of systemic arthralgia persisting for 2 weeks. His condition spontaneously improved; however, bilateral hearing loss subsequently developed. The patient exhibited weight loss of 10 kg within 1 month. Therefore, he was admitted to Fukuoka University Hospital for a further examination. His laboratory data revealed elevated serum levels of LDH (200 IU/l), ferritin (70,000 ng/ml), GOT (308 IU/l) and GPT (236 IU/l), suggesting hepatic damage. FDG-PET showed a high FDG uptake in the lymph nodes throughout the body, spleen and right femoral bone marrow (Fig. 6a). The level of cerebral glucose metabolism was decreased in the bilateral parieto-occipital association cortices (Fig. 6b–d). The blood sugar level was 85 mg/dl, and subsequent brain MRI did not show any abnormalities (Fig. 6e). A bone marrow biopsy revealed the involvement of T-cell lymphoma. Following the administration of chemotherapy with CHOP, the patient’s clinical symptoms and laboratory data markedly improved. Post-treatment FDG-PET/CT showed no areas of abnormal uptake in the systemic lymph nodes, spleen and bone marrow (Fig. 7a) with improved cerebral glucose metabolism (Fig. 7b–d).

## Discussion

In the present study, we confirmed the findings of a report by Hanaoka et al. [2] that indicated that the mean cerebral SUV of FDG is correlated with the total glycolytic volume. In addition, we demonstrated that the regional cerebral glucose metabolism changes in association with the TGV in patients with malignant lymphoma of the body, namely, a negative correlation between the degree of cerebral glucose metabolism and the total glycolytic volume was observed in the bilateral frontal, temporal and parietal cortices. After chemotherapy, this finding improved in the good responders. The comparison of regional cerebral glucose metabolism between normal control subjects and the pre-treatment patients also showed similar results. Namely, regional cerebral glucose metabolism decreased in the parietal and occipital regions in patients with lymphoma, although the significant level was lower in the latter. These results highly suggest that regional cerebral metabolic changes are caused by the presence of malignant lymphoma lesions. To the best of our knowledge, this is the first report of regional cerebral metabolic changes in patients with malignant lymphoma of the body evaluated using statistical image analysis.

Several explanations can be considered regarding the mechanisms underlying the regional decrease in cerebral glucose metabolism. One of the most plausible mechanisms is the presence of intravascular involvement of lymphoma cells, although only one patient of 30 patients was diagnosed with IVL. IVL is a relatively rare clinical entity [6] characterized by occlusion of the arterioles, capillaries and venules due to the proliferation of malignant lymphomatous cells [6, 7]. According to the WHO classification, this condition is a subtype of diffuse large-cell lymphoma [8]. When the central nervous system is involved, clinical symptoms, such as stroke or dementia, may occur. The parietal cortices are located in the distal portion of the middle cerebral arteries and posterior cerebral arteries, so-called “watershed areas”. Occlusion of the small arteries may induce ischemia in neurons and neuronal dysfunction, as well as decreased cerebral glucose metabolism. However, almost all cases in previous reports of central nervous system involvement of IVL were associated with neurological deficits or organic lesions on CT/MR images. As shown in the case presentation section (Figs. 4, 5), the degree of cerebral glucose metabolism decreased without apparent cerebral lesions on MRI in this study. Regarding the FDG-PET findings of malignant lymphoma, it is well known that primary cerebral lymphoma exhibits a higher FDG uptake in the lesions than in the cerebral cortices [9], and it has also been reported that cerebral involvement of malignant lymphoma is associated with a high FDG uptake [10]. If the decrease in cerebral glucose metabolism is caused by IVL involvement, it may be possible to successfully demonstrate functional alternations of the brain in the preclinical stage of intravascular involvement with lymphoma cells.

Another possible mechanism is an auto-immunogenic reaction known as paraneoplastic syndrome [7]. PNS is detected in less than 0.1 % of patients with malignant tumors [11], such as small-cell lung cancer, thymoma, breast cancer, ovarian cancer and malignant lymphoma. A diagnosis of PNS is confirmed when the symptoms disappear following the removal or shrinkage of the tumor [12]. Voltz [13] reported that cerebellar glucose metabolism is decreased in patients with PNS. PNS is also caused by cytokines secreted from the activated of immune system cells, such as natural killer cells, due to the existence of tumor cells. Regarding this point, the brain function and metabolism are affected in patients with autoimmune diseases, such as Hashimoto’s or Basedow’s disease [14]. As shown in case 2, cerebral metabolic abnormalities can occur in patients with malignant lymphomas other than intravascular lymphomas. Therefore, this mechanism may function in cases of malignant lymphoma. The parietal and occipital association cortices play an important role as the center of visuospatial recognition. These areas share many neuronal connections with other parts of the brain via synaptic transmission. Antibodies associated with tumors may induce cross talk with neuronal structures, such as synapses or axons. These areas are frequently affected in patients with Alzheimer’s disease [15]; thus, involvement of the parietal cortices may cause dementia. As no patients presented with apparent symptoms of dementia in the present study and the decrease in cerebral glucose metabolism caused by Alzheimer’s disease is usually progressive and irreversible, it is unlikely that the decrease in cerebral glucose metabolism is caused by occasional coincident dementia.

Cerebral glucose metabolism is affected by the mental state of the patient. Tashiro et al. [16] reported that pre-treatment cancer patients exhibit reduced cerebral glucose metabolism in the prefrontal area, lateral frontal area, anterior cingulate gyrus, insular cortex and putamen and that a depressive state may be related to metabolic abnormalities in these regions. The authors suggested that brain PET images may help to diagnose psychoneurological conditions in cancer patients. It has also been reported that approximately 50 % of cancer patients have neuropsychiatric problems fulfilling the DSM-III or DSM-R criteria III [17, 18], and that the prevalence of a depressive state in cancer patients ranges from 1.5 [19] to 53 % [20]. Although we were unable to precisely evaluate the mental state of the patients with malignant lymphoma due to the limitations of the study design, a subclinical depressive state may affect cerebral glucose metabolism. In the present study, cerebral glucose metabolism was decreased in the bilateral cerebellar hemisphere, right putamen, bilateral insula and bilateral anterior cingulate regions after chemotherapy. This reduction in cerebellar and putamen glucose metabolism may merely reflect the mirror effect of improved cerebral glucose metabolism. Meanwhile, the decreased glucose metabolism in the insula and anterior cingulate gyrus may be related to the mental state of the patients. On the other hand, it is well known that chemotherapy using anticancer drugs can result in brain damage, such as leukoencephalopathy [21]. We found no apparent evidence of leukoencephalopathy in our patients; however, some cases of brain damage may have occurred with subsequent decreased cerebral glucose metabolism.

The plasma glucose level may change the regional distribution of FDG in the brain as well as the global brain FDG uptake. Kawasaki et al. reported that the FDG distribution pattern is altered under conditions of mild hyperglycemia and that a decreased uptake pattern resembles to that observed in patients with Alzheimer’s disease. However, in the present study, there were no significant differences in the plasma glucose levels between the pre- and post-treatment examinations; hence, it is thought to be unlikely that a decreased regional cerebral glucose metabolism is caused by changes in the plasma glucose levels in patients with malignant lymphoma. Regarding the effect of FDG shift from the brain to the huge tumor burden, this phenomenon reduces the total amount of FDG in the brain, but it is not considered to affect the regional distribution of FDG in the brain, because the dose of the carrier FDG is very small (≪1 mg).

The limitations of this study include the following. As this study was retrospectively performed referring to medical records, the subjects’ mental state and function were not evaluated objectively. Organic brain lesions were evaluated primarily using CT with low-dose PET/CT examinations, except in a small number of patients. Therefore, some organic lesions of the brain may not have been detected. In addition, normal control subjects were neither age matched nor sex matched due to limited number of normal control subjects at our institute. This may affect the results of the present study. Regarding the cerebral glucose metabolism, the absolute values of cerebral glucose metabolism were not evaluated in this study, because it required invasive techniques such as arterial blood sampling. Thus, our findings of decrease in regional cerebral glucose metabolism were just relative ones.

In conclusion, we herein demonstrated that patients with malignant lymphoma of the body exhibit abnormal regional cerebral glucose metabolism that improves after chemotherapy. Although the mechanism underlying the reduction in cerebral glucose metabolism remains unclear, precise observations of brain data extracted from whole-body scans may reveal functional alternation or subclinical damage of the brain in patients with malignant lymphoma that may have been overlooked in whole-body FDG-PET/CT studies.
